# Efficacy and Safety of Mitoxantrone Hydrochloride Injection for Tracing Axillary Sentinel Nodes in Breast Cancer: A Self-Controlled Clinical Trial

**DOI:** 10.3389/fonc.2022.914057

**Published:** 2022-06-08

**Authors:** Dechuang Jiao, Benlong Yang, Jiajian Chen, Chunjian Wang, Lidan Jin, Wenhe Zhao, Xueqiang Gao, Haibo Wang, Jun Li, Haidong Zhao, Di Wu, Zhimin Fan, Shujun Wang, Zhenzhen Liu, Yongsheng Wang, Jiong Wu

**Affiliations:** ^1^ Department of Breast Disease, Henan Breast Cancer Center, The Affiliated Cancer Hospital of Zhengzhou University & Henan Cancer Hospital, Zhengzhou, China; ^2^ Department of Breast Surgery, Fudan University Shanghai Cancer Center, Shanghai, China; ^3^ Department of Oncology, Shanghai Medical College of Fudan University, Shanghai, China; ^4^ Department of Collaborative Innovation Center for Cancer Medicine, Fudan University, Shanghai, China; ^5^ Department of Breast Surgery, Shandong Cancer Hospital Fudan University, Jinan, China; ^6^ Department of Oncological Surgery, Affiliated Sir Run Run Shaw Hospital, Zhejiang University School of Medicine, Hangzhou, China; ^7^ Department of Breast Surgery, The Affiliated Hospital of Qingdao University, Qingdao, China; ^8^ Department of Breast Surgery, Second Affiliated Hospital of Dalian Medical University, Dalian, China; ^9^ Department of Breast Surgery, The First Bethune Hospital of Jilin University, Changchun, China; ^10^ Department of Pharmaceutics, School of Pharmacy, Shenyang Pharmaceutical University, Shenyang, China

**Keywords:** breast cancer, sentinel lymph node biopsy, mitoxantrone hydrochloride injection for tracing, mastectomy, breast-conserving surgery, radioactive tracers

## Abstract

**Background:**

Mitoxantrone hydrochloride injection for tracing (MHI), a new strategy to identify lymph nodes, has not been tested for axillary node staging in breast cancer. This multicenter, self-controlled, non-inferiority trial aimed to evaluate MHI’s efficacy and safety in sentinel lymph node biopsy (SLNB).

**Methods:**

The trial was conducted across seven hospitals from December 2019 to December 2020. Patients with early-stage breast cancer received MHI and technetium-99m (99mTc) during the surgery. Sentinel node detection rates were compared between MHI and 99mTc to evaluate non-inferiority and concordance. Non-inferiority was valid if the lower limit of the 95% CI of sentinel node relative detection rate difference was ≥−5%.

**Results:**

SLN relative detection rate of MHI was 97.31% (362/372). Of the SLNs, 79.69% (871/1093) were co-detected by both tracers. Of the patients, 4.13% (16/387) had adverse events and recovered during the follow-up.

**Conclusions:**

MHI is a lymphatic tracer with comparable efficacy to radionuclides and can be used alone or in combination with radioactive substances for SLNB.

**Clinical Trial Registration:**

http://www.chinadrugtrials.org.cn, CTR20192435.

## Introduction

In 2020, breast cancer surpassed lung cancer as the leading contributor to the global cancer incidence, with an estimated 2.3 million new cases, representing 11.7% of all cancer cases (1). The number of new cases of female breast cancer in China is 416,371. The incidence of breast cancer in China ranks fourth among all cancers, but the number of patients with breast cancer in China ranks first in the world (2). Sentinel lymph node biopsy (SLNB) is the current standard care for axillary node staging in clinically axillary node-negative breast cancer patients. SLNB is equivalent to axillary lymph node dissection (ALND) in terms of accurate staging, prognosis, and treatment guidance, and it offers the advantages of lower morbidity rates and fewer complications than ALND, including less upper limb lymphedema and fewer shoulder joint movement disorders (3). Lymphatic mapping and SLNB have improved the ability of surgeons to detect small-volume disease in lymph nodes while greatly reducing intraoperative and postoperative morbidity. Large randomized controlled trials have shown SLNB to be a robust method, with detection rates of >97% and low false-negative rates (FNRs) (4).

Although the gold standard for the detection of SLN is a radioactive tracer combined with blue dye (5), the accessibility of this procedure is only 60% in developed countries, with this figure dropping down to 5% in China and even lower in the rest of the world (6). In a survey (7), methylene blue is the most commonly used SLN tracer in China, and the availability of radioactive colloids is low. Methylene blue has good visibility, is cheap, and is easy to obtain. However, its lymphatic targeting is poor, and it is easy to infect secondary lymph nodes and can cause allergic reactions such as skin rash. The carrier sulfur colloid of radionuclides is not available for most of the hospitals in China. Nuclear medicine facilities are not available at most centers, and the hand-held gamma probe that is used for intraoperative SLNB is expensive. Some members of the medical staff have concerns about radioactive colloids. Thus, there was a long felt need to investigate further to identify a cost-effective and accurate method of SLNB.

These limitations have motivated studies on novel methods to guide SLNB, such as indocyanine green (ICG) optical imaging (8) and superparamagnetic iron oxide (SPIO) imaging (6), which have high SLN detection rates. Research (9) has also revealed that the detection rate of SLNs with ICG is 94%. Additionally, SPIO is equivalent to the technetium-99m (99mTc) radiotracer for identifying SLNs in breast cancer, as demonstrated by Rubio et al. (10) However, SPIO tracers are not accessible in China, and there are shortcomings in their technology.

Mitoxantrone is an antineoplastic antibiotic commonly used intravenously to treat acute leukemia, lymphoma, prostate cancer, and breast cancer. It is metabolized mainly by the biliary tract. Preclinical pharmacodynamics studies of mitoxantrone hydrochloride have shown that it has a high affinity for the lymphatic system after subcutaneous or subserosal administration. After administration in the interstitial space, mitoxantrone hydrochloride will gradually settle into nanocrystals due to the microenvironmental change in the pH value. The particle size of these nanocrystals is approximately 100 nm (11), and their distribution is uniform. These nanocrystals can enter lymphatic capillaries through the space between endothelial cells or through endocytosis and phagocytosis of these cells, but they cannot enter the blood circulation. (The space between blood capillary endothelial cells is 30–50 nm, but the space between lymphatic capillary endothelial cells is 120–500 nm.) Then, they accumulate in the regional lymph nodes through lymphatic drainage and stay in the lymph nodes for a period of time, staining the lymph nodes blue. Mitoxantrone hydrochloride injection for tracing (MHI) was approved by the NMPA on June 22, 2021 to trace draining lymph nodes in the thyroid surgical region. One study (12) showed that the use of MHI showed ideal lymph node tracing function, along with high safety and good tolerability. In a previous single-center, self-controlled, phase I trial, we evaluated the safety and tolerability of MHI for lymphatic mapping in patients with breast cancer. No drug-limiting toxic effects were observed when the dose was increased to 2.0 ml (5 mg/ml), and there were no significant differences in the detection rates or number of lymph nodes detected by MHI and 2 mCi 99mTc-labeled sulfur colloid (13).

This present phase III trial was designed to establish the non-inferiority of MHI to radioisotopes in SLN detection and to conduct premarket clinical studies for drug registration approval.

## Material and Methods

### Study Design

This was a phase 3, multicenter, self-controlled, non-inferiority study approved by the ethics committee of the lead center (the medical ethics committee of Fudan University Affiliated Tumor Hospital) and the ethics committees of the other six participating centers. All procedures were conducted in accordance with the tenets of the Declaration of Helsinki. All participants signed the informed consent form prior to any study procedure. 99mTc-labeled sulfur colloid was used as the standard to evaluate the efficacy and safety of MHI for tracing axillary sentinel nodes in patients with early-stage breast cancer. The number of stained or imaged lymph nodes and pathological examination results were recorded. Electrocardiogram, laboratory tests (e.g., blood routine, blood biochemistry, urine routine, and coagulation function), and other related tests were performed for safety assessment during the screening period, namely, (−28 to −1 days), 3 ± 1 days, and 14 ± 3 days postoperative, respectively.

### Participants

This study included patients with a diagnosis of primary invasive breast cancer or ductal carcinoma *in situ* (DCIS) who were scheduled to undergo SLNB. Patients were required to be 18–70 years of age, with clinically T1–3 and node-negative axilla. Patients had no severe hematological, hepatic, or renal dysfunction. The exclusion criteria were open biopsy, a history of breast augmentation, previous breast cancer surgery or neoadjuvant chemotherapy, a need for bilateral breast cancer surgery, infectious diseases (e.g., HVB, HIV, and HCV), pregnancy, or lactation. Additionally, patients who had participated in any other clinical trials within 4 weeks prior to the study or were considered inappropriate to participate in the trial by the investigator were excluded. Patients had the right to withdraw from the trial at any time.

### Surgical Procedure

Each patient received an injection of 2 mCi 99mTc-labeled sulfur colloid (as a self-control) into the breast tissue surrounding the tumor 24 h before surgery. MHI (5 mg/ml) was injected into the same site approximately 15 min before dissection. The total dose did not exceed 2.0 ml. There were multiple sites of subcutaneous injections of MHI in the areola and around the tumor in mastectomy patients and around the tumor in breast-conserving patients. Manual kneading of the breast was performed for 5 min following the injection. All stained lymph nodes and/or lymph nodes with stained lymphatic vessels were considered as SLNs. These SLNs were visualized with blue staining and excised first. Excised blue-stained SLNs were then tested for radioactivity using a gamma-detecting probe and classified as “hot spot” or “no hot spot.” Finally, the gamma-detecting probe was used to find and removed the rest radioactivity “hot spot” in the breast. SLN sectioning and processing for histopathological examination were performed using standard protocols. The pathological status of each detected lymph node was determined by intraoperative imprint cytology and/or frozen sectioning.

### Study Endpoints and Definitions

All patients with hot spots were included in the analysis, and the relative detection rate of MHI was calculated.

The primary endpoint was the SLN relative detection rate of MHI. The SLN relative detection rate was calculated as follows: (number of patients detected by MHI/number of patients detected by 99mTc) × 100%. A patient detected by MHI refers to a patient with stained lymph nodes and/or lymph nodes with stained lymphatic vessels. A patient detected by 99mTc refers to a patient with hot spots in the lymph nodes (the highest gamma probe count or the count value was no less than 10% of the highest count).

The secondary endpoints were the consistency between MHI and 99mTc. Tracing consistency was calculated as follows: (number of SLNs detected by MHI and 99mTc simultaneously/number of SLNs detected by 99mTc) × 100%. Descriptive statistical analysis was performed on the total number of SLNs, the number of identical SLNs detected by two tracers, and the number of patients with detected SLNs. Relative detection rate of positive patients was calculated as follows: (number of positive patients detected by MHI and 99mTc/number of positive patients detected by 99mTc) × 100%. A positive patient refers to a patient with at least one positive SLN detected.

Safety was analyzed among patients who received at least one study drug (control drug or test drug) and had a post-baseline safety evaluation. Adverse events (AEs) were categorized according to the Common Terminology Criteria for Adverse Events (CTCAE) version 5.0. The incidence of patients who experienced AEs were calculated. If one patient experienced multiple AEs, it was recorded once when calculating the incidence of subjects experiencing at least one AE.

### Statistical Methods

SAS software, version 9.4 (SAS Institute Inc.) was used for statistical analyses. Generally, continuous variables are expressed as the mean, standard deviation, median, minimum, and maximum. Categorical variables are expressed as frequencies and percentages. The Clopper–Pearson method was used to calculate the 95% confidence interval (CI) of the SLN relative detection rate. The difference between the two arms and its 95% CI were calculated by the Wald method, and the approximate normal test was used to calculate the p-value. Non-inferiority was achieved when the lower limit of the 95% CI for the SLN relative detection rate was greater than or equal to −5%.

### Sample Size and Non-inferiority Margin

We hypothesized that MHI would be non-inferior to 99mTc in the detection of SLNs. The one-sided significance level was 2.5%, the non-inferiority margin was 5%, and the power of this study was 90%. Under this assumption, a sample size of 323 patients was required to show non-inferiority of MHI. Considering the dropout rate, the final sample size of this study was fixed at 388 enrolled patients.

## Results

### Patient Disposition

**Figure 1:**
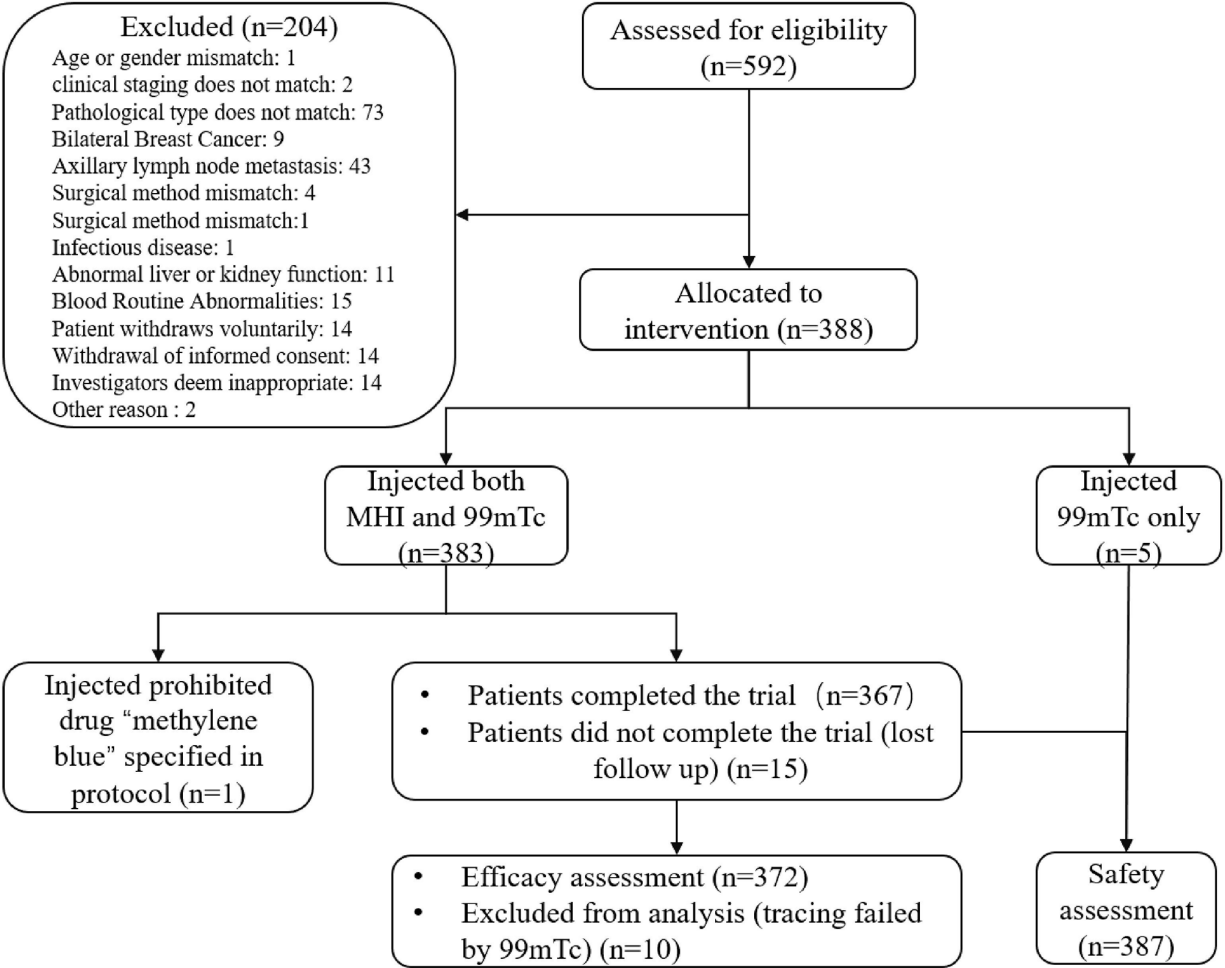
Patient disposition. A total of 592 patients were screened; 204 failed screening, and 388 were ultimately enrolled. Of the enrolled patients, 383 were injected with both MHI and 99mTc. One patient was not included in the FAS or SS due to the use of the prohibited drug “methylene blue” as specified in the protocol. At the discretion of the Data Review Meeting, 382 patients were included in the FAS, and 387 patients were included in the SS.

In total, 592 patients were screened between December 2019 and December 2020. Among them, 204 failed screening, and 388 patients were enrolled the study. Three hundred eighty-three patients were injected with both MHI and 99mTc. Three hundred sixty-seven patients completed the trial, and 21 withdrew prematurely (**Figure 1**). Among these patients, one was not included due to the use of the prohibited drug “methylene blue” specified in the protocol. Based on Chinese medical resources, patient wishes, and follow-up conditions, 221 (57.53%) patients underwent total mastectomy, and 161 (42.47%) patients underwent breast-conserving surgery. General patient characteristics are listed in **Table 1**.

**Table 1:** General patient characteristics.

	n = 372
Age	52.4 (26–70)
Body mass index	23.84 (16.58–38.77)
Tumor type	
Invasive breast cancer	327 (87.90%)
Ductal carcinoma *in situ*	45 (12.10%)
Pathologic tumor stage	
Ti	45 (12.10%)
T1	190 (51.08%)
T2	134 (36.02%)
T3	3 (0.81%)
Procedure	
Breast conservation	158 (42.47%)
Total mastectomy	214 (57. 53%)
Tumor site	
Upper inner	102 (27.42%)
Lower inner	29 (7.80%)
Upper outer	172 (46.24%)
Lower outer	40 (10.75%)
Central area	8 (2.15%)
Unknown	21 (5.65%)

### Primary Endpoint

**Figure 2:**
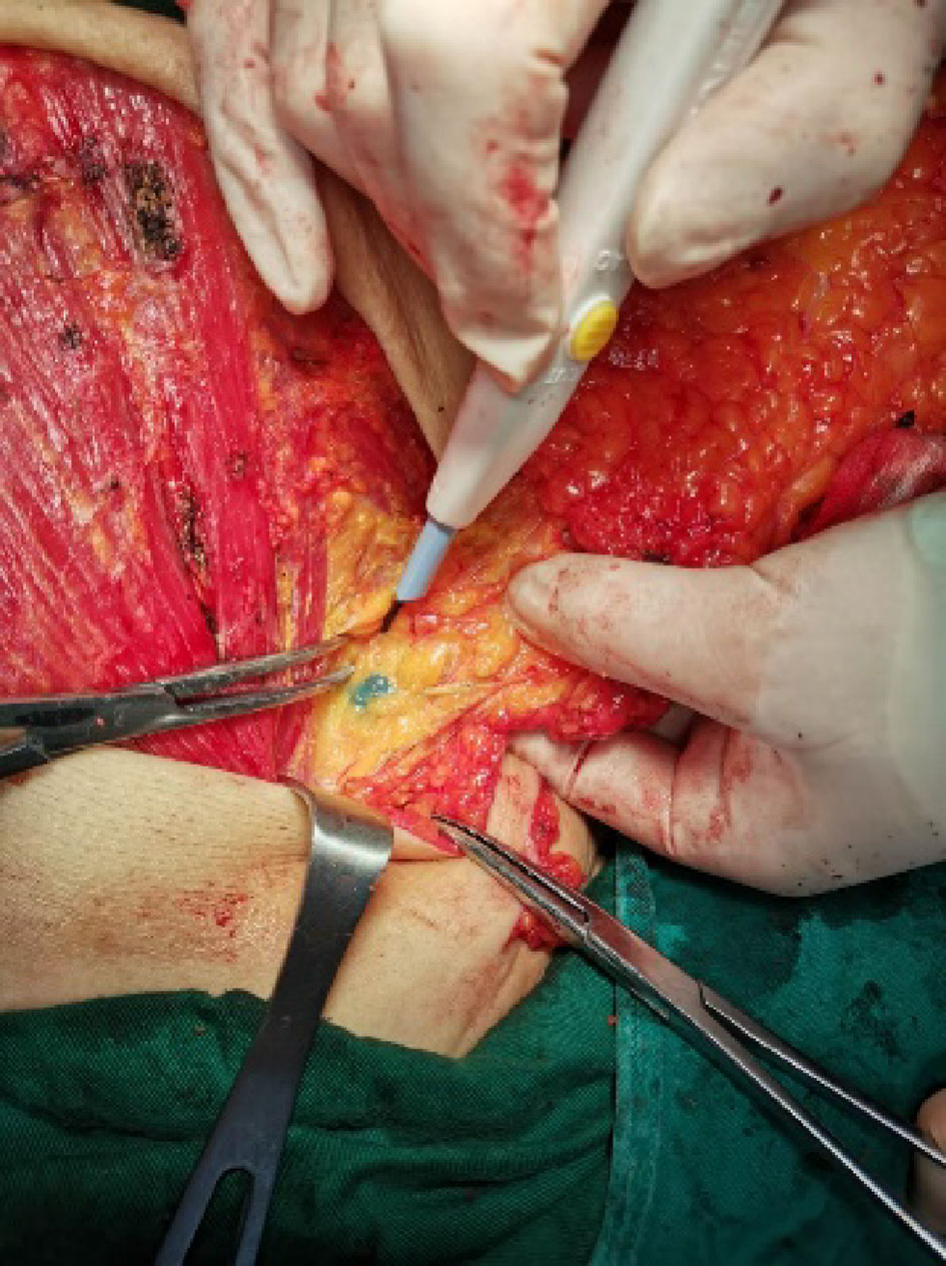
SLN detected by MHI. Lymph nodes were stained blue by MHI and could be clearly distinguished from surrounding tissue.

Three hundred seventy-two patients had hot spots detected by 99mTc. Three hundred sixty-two of them were identified by MHI (**Figure 2**). The SLN relative detection rate (and 95% CIs) of MHI was 97.31% (95.11%, 98.70%). The difference (and 95% CI) in the SLN relative detection rates of MHI and 99mTc was −2.69% (−4.33%, −1.04%). Since the lower limit of the 95% CI was greater than −5%, the SLN relative detection rate of MHI was non-inferior to that with 99mTc.

### Secondary Endpoints

#### Tracing Consistency

A total of 1,093 SLNs were detected by 99mTc. The average number of SLNs detected by 99mTc was 2.9 ± 1.73, the median number of SLNs detected was 2.5, and the number of SLNs detected ranged from 1 to 12. 871 of those SLNs identified by MHI. The average number of SLNs detected by MHI was 2.9 ± 1.81, the median number of SLNs detected was 3.0, and the number of SLNs detected ranged from 1 to 13. The tracing consistency between MHI and 99mTc was 79.69% (871/1093), and 95% CIs of tracing consistency were 77.18%–82.04%.

#### Relative Detection Rate of Positive Patients

Based on the analysis of 372 subjects, 80 patients were detected with positive SLNs by 99mTc, of which 75 patients were also detected with positive SLNs by MHI. The relative co-detection rate of positive patients was 93.75% (75/80).

### Safety

Of the patients, 4.1% (16/387) experienced AEs that might have been related to study drugs (**Table 2**). The main AE was abnormal liver function tests. All of them recovered at follow-up.

**Table 2:** Patients experiencing adverse events that possibly related to study drugs.

System organ class-preferred term	Number of patients (n = 16)	Number of times			
		Grade 1	Grade 2	Grade 3	Total
All adverse events	16 (4.1%)				
Infections and infestations	1 (0.3%)				
Papulopustular rash	1 (0.3%)	1	0	0	1
Investigations	16 (4.1%)				
ALT increased	16 (4.1%)	9	5	7	21
AST increased	15 (3.9%)	9	3	4	16
GGT increased	5 (1.3%)	2	3	1	6
ALP increased	2 (0.5%)	2	0	0	2

ALT, alanine aminotransferase; AST, aspartate aminotransferase; GGT, gamma-glutamyltransferase; ALP, blood alkaline phosphatase

## Discussion

Successful SLNB depends in part on the lymphatic tracer. Li et al. (14) reported that the SLN detection rate of methylene blue was 91%, and the FNR was 13%. Studies (15) have shown a high SLN detection rate of patent blue (>95%) and low FNRs of 5%–10%. Guo et al. (16) reported that the SLN detection rate of ICG alone was 97%, and the SLN detection rate of ICG combined with methylene blue was 99.5%. This study evaluated the SLN relative detection rate of MHI as a new dye tracer. The SLN relative detection rate of MHI reached 97.31%, and its non-inferiority to 99mTc was established. Besides, nine positive patients were detected by MHI but not by 99mTc. MHI had detected more positive SLNs but less total SLNs than 99mTc. This may indicate that the accuracy and FNR of MHI were no worse than 99mTc in SLNB.

The main adverse reactions to mitoxantrone use in chemotherapy are nausea and vomiting, stomatitis, and alopecia, and the majority of these cases are mild. Many patients experience no adverse reactions to mitoxantrone (17). There are few reports of cellulitis, vesication, or tissue necrosis following extravasation. When used as an SLN tracer, MHI can enter the lymphatic capillaries and be enriched in regional lymph nodes but has difficulty entering the blood circulation. MHI is removed with the lymph nodes during surgery. In a previous phase I study (13), the C_max_ detected in the plasma ranged between 26 and 79.4 ng/ml for patients receiving a 2.0-ml dose of MHI. Thus, systemic side effects are mild. The most frequent AE related to MHI in this study was an abnormal biochemical indicator related to liver function, which is related mainly to the metabolic pathway of the drug and may suggest that we should use this product with caution for SLN tracing in patients with abnormal liver function or previous liver damage. However, it should be noted that all of these patients recovered after symptomatic treatment. Nearly half of the patients in this study underwent breast-conserving surgery. The protocol specified that for breast-conserving surgery, the injection site was at the peritumoral site, generally in the gland. By the end of the follow-up period, there were no AEs, such as skin necrosis at the injection site. In this study, one patient who underwent breast reconstruction surgery experienced postoperative wound infection, but this AE was unrelated to MHI.

MHI and 99mTc have different identification methods for SLN tracing. This study conducted MHI tracing first and then conducted 99mTc tracing so that we can avoid the over-valuation of MHI. This study also has some limitations. ALND could not be performed on all patients to assess the SLN false-negative rate for ethical consideration. The study also did not include patients who underwent excisional biopsy and neoadjuvant chemotherapy, in which the accuracy of the use of the new tracer remains to be confirmed by further studies.

## Conclusions

This prospective multi-institutional study showed MHI for tracing to be an another ideal tracer with efficacy and safety that can be used alone or combined with radioactive material. We expect that the application of MHI in the field of breast cancer can improve the accessibility of SLNB and bring good prognosis to more patients.

## Data Availability Statement

The raw data supporting the conclusions of this article will be made available by the authors, without undue reservation.

## Ethics Statement

The studies involving human participants were reviewed and approved by Medical Ethics Committee of Fudan University Affiliated Tumor Hospital; Ethics Committee of Drug Clinical Trials, Shandong Cancer Hospital; Medical Ethics Committee of Affiliated Hospital of Qingdao University; Medical Ethics Committee of Run Run Shaw Hospital Affiliated to Zhejiang University School of Medicine; Ethics Committee of the First Hospital of Jilin University; Medical Ethics Committee of Henan Cancer Hospital; and Neighborhood Committee of the Second Affiliated Hospital of Dalian Medical University. The patients/participants provided their written informed consent to participate in this study.

## Author Contributions

DJ, BY, JC, and CW contributed to investigation, formal analysis, and writing—original draft. LJ, XG, JL, and DW contributed to investigation and data curation. WZ, HW, HZ, and ZF contributed to resources, supervision, and project administration. SW contributed to methodology and conceptualization. ZL, YW, and JW contributed to conceptualization, methodology, resources, writing—review and editing, and funding acquisition. All authors contributed to the article and approved the submitted version.

## Funding

This work was supported by grants from the National Key R&D Program of China (2017YFC1311004) and National Natural Science Foundation of China (81672638, 81502314, 81302297, and 81973494).

## Conflict of Interest

The authors declare that the research was conducted in the absence of any commercial or financial relationships that could be construed as a potential conflict of interest.

The reviewer XW declared a shared affiliation with the authors CW and YSW to the handling editor at the time of review.

## Publisher’s Note

All claims expressed in this article are solely those of the authors and do not necessarily represent those of their affiliated organizations, or those of the publisher, the editors and the reviewers. Any product that may be evaluated in this article, or claim that may be made by its manufacturer, is not guaranteed or endorsed by the publisher.

## References

[1] Sung H Ferlay J Siegel RL Laversanne M Soerjomataram I Jemal A. Global Cancer Statistics 2020: GLOBOCAN Estimates of Incidence and Mortality Worldwide for 36 Cancers in 185 Countries. CA Cancer J Clin (2021) 71:209–49. doi: 10.3322/caac.21660 33538338

[2] Zhang Y Lu Z Song F Chen K. Trends in the Incidence and Mortality of Breast Cancer Globally and in My Country. J Multidiscip. Cancer Manag (2021) 7:14–20.

[3] Veronesi U Paganelli G Viale G Luini A Zurrida S Galimberti V. A Randomized Comparison of Sentinel-Node Biopsy With Routine Axillary Dissection in Breast Cancer. N Engl J Med (2003) 349:546–53. doi: 10.1056/NEJMoa012782 12904519

[4] Kim T Giuliano AE Lyman GH. Lymphatic Mapping and Sentinel Lymph Node Biopsy in Early-Stage Breast Carcinoma: A Metaanalysis. Cancer (2006) 106:4–16. doi: 10.1002/cncr.21568 16329134

[5] Giuliano AE Hunt KK Ballman KV Beitsch PD Whitworth PW Blumencranz PW. Axillary Dissection vs No Axillary Dissection in Women With Invasive Breast Cancer and Sentinel Node Metastasis: A Randomized Clinical Trial. JAMA (2011) 305:569–75. doi: 10.1001/jama.2011.90 PMC5389857 21304082

[6] Karakatsanis A Christiansen PM Fischer L Hedin C Pistioli L Sund M. The Nordic SentiMag Trial: A Comparison of Super Paramagnetic Iron Oxide (SPIO) Nanoparticles Versus Tc(99) and Patent Blue in the Detection of Sentinel Node (SN) in Patients With Breast Cancer and a Meta-Analysis of Earlier Studies. Breast Cancer Res Treat (2016) 157(2):281–94. doi: 10.1007/s10549-016-3809-9 PMC4875068 27117158

[7] Yang B Ren G Song E Pan D Zhang J Wang Y. Current Status and Factors Influencing Surgical Options for Breast Cancer in China: A Nationwide Cross-Sectional Survey of 110 Hospitals. Oncologist (2020) 25:e1473–80. doi: 10.1634/theoncologist.2020-0001 PMC7543333 32333626

[8] Staubach P Scharl A Ignatov A Ortmann O Inwald EC Hildebrandt T. Indocyanine Green (ICG) Fluorescence Imaging Versus Radioactive Colloid for Sentinel Lymph Node Identification in Patients With Breast Cancer. J Clin Oncol (2010) 28:674. doi: 10.1200/jco.2010.28.15_suppl.674

[9] Kitai T Inomoto T Miwa M Shikayama T. Fluorescence Navigation With Indocyanine Green for Detecting Sentinel Lymph Nodes in Breast Cancer. Breast Cancer (2005) 12:211–5. doi: 10.2325/jbcs.12.211 16110291

[10] Rubio IT Diaz-Botero S Esgueva A Rodriguez R Cortadellas T Cordoba O. The Superparamagnetic Iron Oxide is Equivalent to the Tc99 Radiotracer Method for Identifying the Sentinel Lymph Node in Breast Cancer. Eur J Surg Oncol (2015) 41:46–51. doi: 10.1016/j.ejso.2014.11.006 25466980

[11] Mao Y Liu J Shi T Chen G Wang S. A Novel Self-Assembly Nanocrystal as Lymph Node-Targeting Delivery System: Higher Activity of Lymph Node Targeting and Longer Efficacy Against Lymphatic Metastasis. AAPS PharmSciTech (2019) 20:292. doi: 10.1208/s12249-019-1447-3 31428888

[12] Chen S Liu W Zhang S Sun J Yang R Wang S. Clinical Efficacy, Safety and Pharmacokinetics of Tracing Injection of Mitoxantrone Hydrochloride for Tracing Sentinel Lymph Nodes in Thyroid Carcinoma: A Phase I Clinical Trial. Med J Peking Union Med Coll Hosp (2021) 12(5):729–35. doi: 10.12290/xhyxzz.2021-0281

[13] Yang B Zheng S Huang X Chen J Liu Z Liu G. A Single-Center, Self-Controlled, Phase I Clinical Trial of Mitoxantrone Hydrochloride Injection for Lymph Tracing for Sentinel Lymph Node Identification of Breast Cancer. Gland Surg (2021) 10:992–1001. doi: 10.21037/gs-20-694 33842243 PMC8033053

[14] Li J Chen X Qi M Li Y. Sentinel Lymph Node Biopsy Mapped With Methylene Blue Dye Alone in Patients With Breast Cancer: A Systematic Review and Meta-Analysis. PloS One (2018) 13:e0204364. doi: 10.1371/journal.pone.0204364 30235340 PMC6147575

[15] Vidya R Athwal R Huissoon AP Baretto RL Krishna MT. Diagnostic Application of Patent Blue V in Sentinel Lymph Node Biopsy for Breast Cancer - Is it Time for a Change? Indian J Cancer (2019) 56:269–70. doi: 10.4103/ijc.IJC_139_18 31389393

[16] Guo J Yang H Wang S Cao Y Liu M Xie F. Comparison of Sentinel Lymph Node Biopsy Guided by Indocyanine Green, Blue Dye, and Their Combination in Breast Cancer Patients: A Prospective Cohort Study. World J Surg Oncol (2017) 15:196. doi: 10.1186/s12957-017-1264-7 29096643 PMC5667473

[17] Crossley RJ. Clinical Safety and Tolerance of Mitoxantrone (Novantrone). Cancer Treat Rev (1983) 10(Suppl B):29–36. doi: 10.1016/0305-7372(83)90019-1 6362875

